# Supplementary material for: Interleukin 10 Restores Lipopolysaccharide-Induced Alterations in Synaptic Plasticity Probed by Repetitive Magnetic Stimulation
Source: Front Immunol. 2020 Dec 16;11:614509. doi: 10.3389/fimmu.2020.614509 (PMC7772211; doi:10.3389/fimmu.2020.614509)
Supplement: Supplementary file 1 [file DataSheet_1.docx]

**SUPPLEMENTARY MATERIAL**

**(Front. Immunol. | doi: 10.3389/fimmu.2020.614509)**


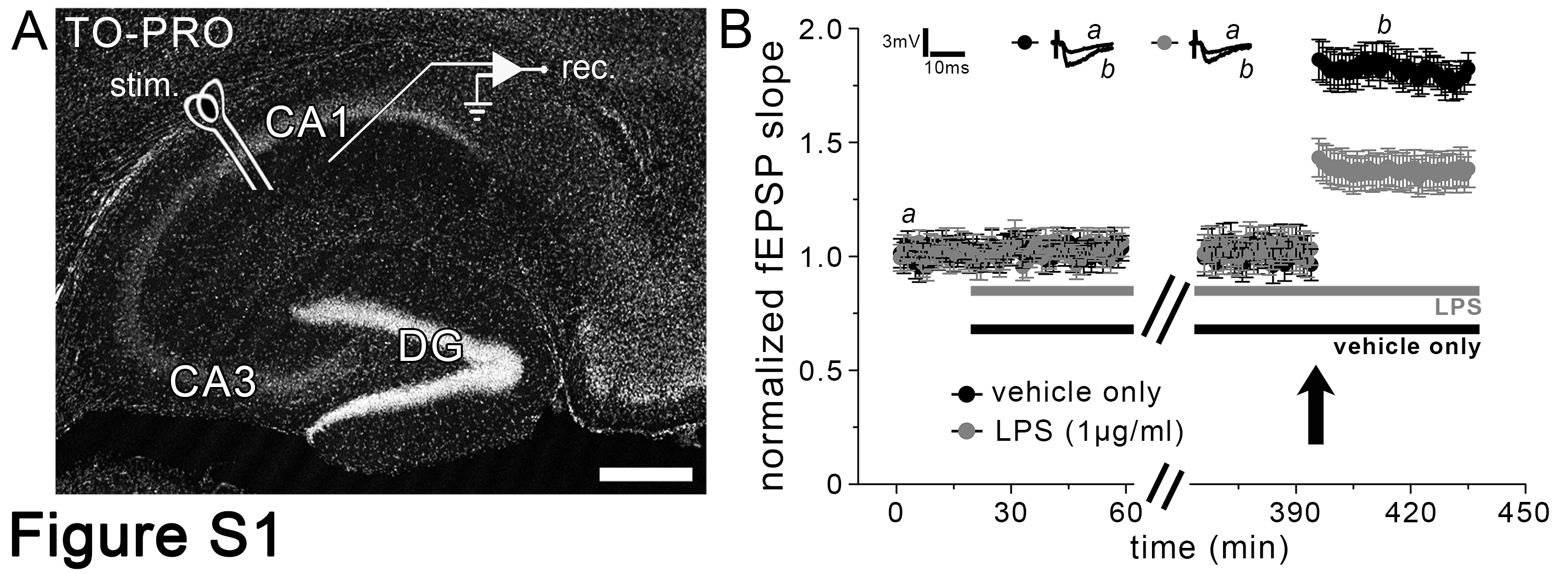


**Figure S1: Long-term potentiation of Schaffer collateral-CA1 synapses is impaired in Lipopolysaccharide-treated acute hippocampal slices.**

**(A, B)** A single 100 Hz electric tetanus (1 s; indicated by arrow in B) is applied to induce LTP at Schaffer collateral-CA1 synapses either in LPS (1 µg/ml) or vehicle-only treated acute hippocampal slices (TO-PRO nuclear stain; Scale bar: 300 μm). While LPS treatment does not affect baseline synaptic transmission, the ability of neurons to express synaptic plasticity is impaired. Representative traces of field excitatory postsynaptic potentials (fEPSP) at indicated times (a, b) are shown on top (n = 12 slices from 4 animals for each group; unpaired two-tailed t-test; p < 0.001).

Values represent mean ± s.e.m.


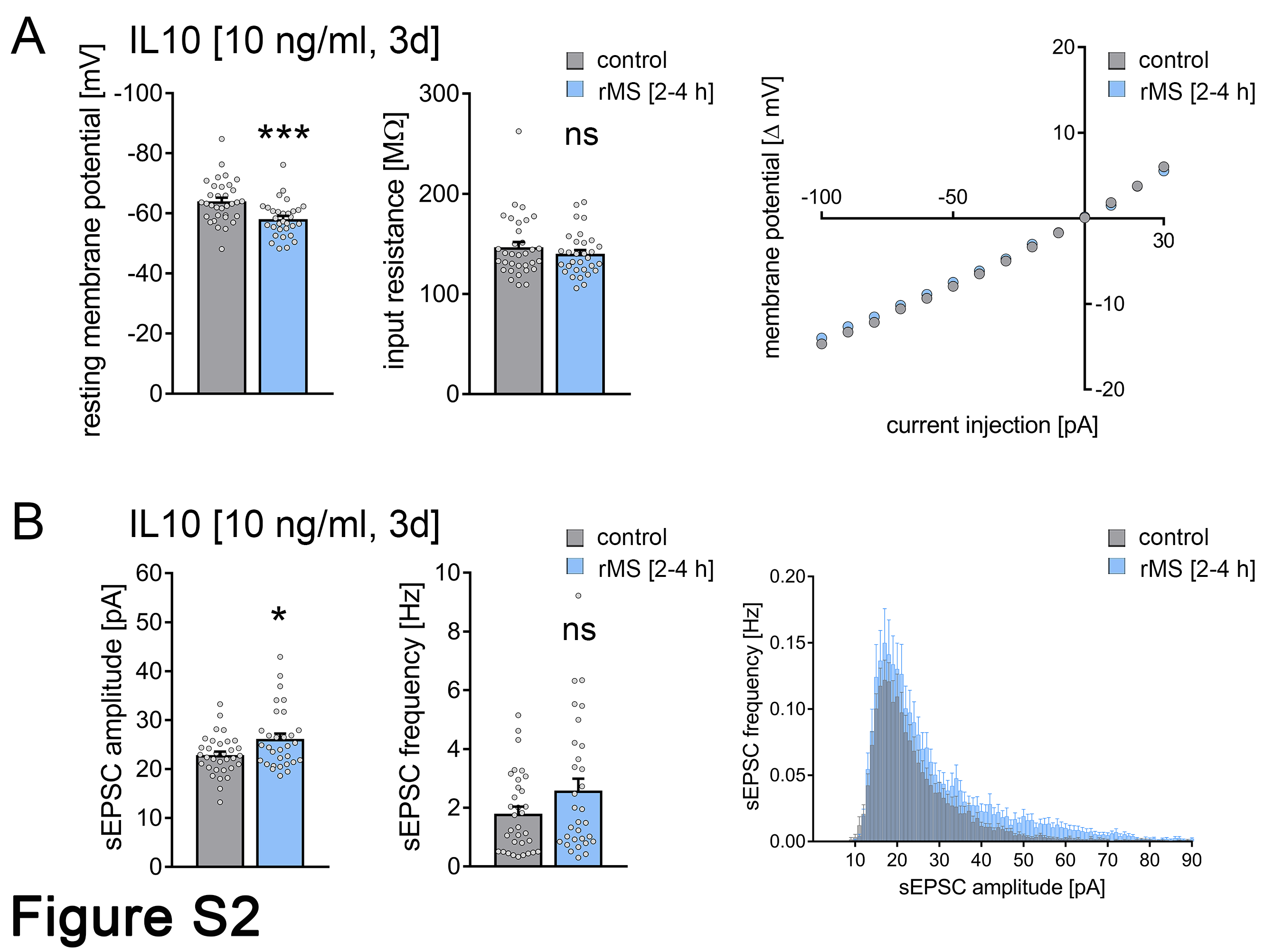


**Figure S2: Interleukin 10 does not occlude rMS-induced synaptic plasticity in CA1 pyramidal neurons of organotypic tissue cultures.**

(A) Tissue cultures were treated with interleukin 10 (10 ng/ml, 3 days) to assess the effects of interleukin-10 pretreatment on rMS-induced synaptic plasticity. Notably, rMS causes a significant increase in the resting membrane potential in these cultures, whereas the input resistance and the input/output-curve do not display significant differences.

(B) rMS-induced strengthening of excitatory synapses in CA1 pyramidal neurons was not hampered by interleukin 10 pretreatment, since sEPSC amplitude but not frequency shows a significant increase upon rMS. (n_control_ = 33 cells in 7 cultures; n_rMS_ = 31 cells in 6 cultures; Mann-Whitney test; U_resting membrane potential_ = 247, U_sEPSC amplitude_ = 363)

Individual data points are indicated by single dots. Values represent mean ± s.e.m. (* p < 0.05, *** p < 0.001; ns, not significant difference).

**Table S1: Primers used in the generation of *C57BL/6-Tg(TNFa-eGFP)* mouse line.**

|  | **Primer** | **Sequence** |
| --- | --- | --- |
| **1a** | RbGLClaIf | gcatatcgatcctgagaacttcagggtga |
| **1b** | RbGL-ERIr | gcatgaattcgccctatagtgagtcgtattaca |
| **2a** | eGFP-ERIf | gcatgaattccaccatggtgagcaagggcga |
| **2b** | eGFP-PAf | ggcatggacgagctgtacaagtaatctagatcataatcagccataccaca |
| **2c** | eGFP-PAr | tgtggtatggctgattatgatctagattacttgtacagctcgtccatgcc |
| **2d** | SV40PA-SalIr | gcatgtcgacttaagatacattgatgagt |
| **3a** | mTNFp-NIBBIf | cagtgcggccgcttcgaagctctaaaagccagccact |
| **3b** | mTNFp-HdIIIr | gcataagcttggtgtcttttctggagggaga |
| **4a** | PGNeoFRT-NheIf | cgatgctagcggggtaaccgaagttcctatactttctag |
| **4b** | PGNeoFRT-SalIr | tggcgtcgactcgcattttgaagttcctattccgaagttcc |
| **5a** | TNFds-NheIf | gcatgctagcgtgatttctgtcttgggatgaagt |
| **5b** | TNFds-KSr | tgacggtacccggggctcttaagacccacttgct |
| **6a** | U1 | ctaggtcccagacacaaagg |
| **6b** | U2 | gatacaagggacatcttccc |
| **6c** | U2a | aagcttggtgtcttttctggagggag |
| **7a** | D1 | atacctagtcattgccttcc |
| **7b** | D2 | acccggtagaattgacctgc |
| **7c** | D2a | ttcccactctgggaattcc |
| **8a** | RP23Southf | atcttctcaacctggatggg |
| **8b** | RP23Southr | atccagccacccaacccc |
| **9a** | RP23SouthUpf | atccccaccagtggcctc |
| **9b** | RP23SouthUpr | atctaattctctcgccatctc |
| **10a** | PR23115f (Seq) | ctaggtcccagacacaaagg |
| **10b** | PR23115r (Seq) | atacctagtcattgccttcc |

Primers used during the generation of the *C57BL/6-Tg(TNFa-eGFP)* mouse strain. Restriction sites used during cloning procedure are underlined. Marked sequences in primers 2b and 2c (red) mark the sequence up- and downstream of the NotI site. Marked sequences in primers 4a and 4b (blue) mark the core of the FRT sequence.

**Table S2: Cq-values for RT-qPCR analysis.**

|  | **vehicle-only** | **LPS** | **LPS + IL10** |
| --- | --- | --- | --- |
| **Figure 3** | | | |
| ***Tnfa*** | 31.67 ± 0.15 | 27.20 ± 0.17 | **-** |
| ***Gapdh*** | 19.42 ± 0.12 | 19.41 ± 0.18 | **-** |
| **Figure 4** | | | |
| ***Tnfa*** | 31.33 ± 0.09 | 27.00 ± 0.35 | 28.62 ± 0.17 |
| ***Gapdh*** | 18.52 ± 0.09 | 18.57 ± 0.12 | 18.92 ± 0.16 |
| **Figure 5** | | | |
| ***Tnfa*** | 25.10 ± 0.23 | 20.12 ± 0.15 | 20.77 ± 0.22 |
| ***Il6*** | 31.24 ± 0.32 | 23.19 ± 0.30 | 26.98 ± 0.33 |
| ***Il1b*** | 25.07 ± 0.41 | 19.03 ± 0.30 | 20.47 ± 0.30 |
| ***Ifng*** | 31.46 ± 0.18 | 32.95 ± 0.33 | 31.15 ± 0.37 |
| ***Il10*** | 33.08 ± 0.20 | 26.52 ± 0.37 | 30.51 ± 0.46 |
| ***Gapdh*** | 13.51 ± 0.13 | 12.56 ± 0.08 | 11.61 ± 0.16 |

Figure 3: n = 18 cultures per group; Figure 4: n = 15-17 cultures per group; Figure 5: n = 6 culture samples per group. Values represent mean ± s.e.m.

**Table S3: Protein levels [pg/ml] from cytokine detection assay.**

|  | **vehicle-only** | **LPS** | **LPS + IL10** |
| --- | --- | --- | --- |
| **TNFα** | 5.29 ± 1.73 | 4073.46 ± 455.09^§^ | 380.95 ± 37.74 |
| **IL6** | 12.54 ± 2.41 | 23241.02 ± 163.27^§^ | 6342.21 ± 1082.86 |
| **IL1β** | 0.05 ± 0.01^*,#^ | 56.41 ± 6.73 | 4.00 ± 0.45 |
| **IFNγ** | 0.02 ± 0.01^*,#^ | 0.60 ± 0.05 | 0.14 ± 0.02 |
| **IL10** | 0.79 ± 0.11^#^ | 205.31 ± 14.75 | 6579.11 ± 148.24^§^ |

n = 6 culturing medium samples each. Values represent mean ± s.e.m.

^*^ two values with non-detectable target protein content (set to “0”)

^#^ values below dynamic detection range

^§^ values above dynamic detection range
